# Theil Entropy as a Non-Lineal Analysis for Spectral Inequality of Physiological Oscillations

**DOI:** 10.3390/e24030370

**Published:** 2022-03-04

**Authors:** Ramón Carrazana-Escalona, Miguel Enrique Sánchez-Hechavarría, Ariel Ávila

**Affiliations:** 1 Department of Basic Sciences, Faculty of Medicine, Universidad de Ciencias Médicas de Santiago de Cuba, Santiago de Cuba 90500, Cuba; ramoncarrazanae@gmail.com; 2 Biomedical Sciences Research Laboratory, Department of Basic Sciences, Faculty of Medicine, Universidad Católica de la Santísima Concepción, Concepción 4090541, Chile; 3 Núcleo Científico de Ciencias de la Salud, Facultad de Ciencias de la Salud, Universidad Adventista de Chile, Chillán 3780000, Chile

**Keywords:** Theil entropy, spectral inequality, frequency spectrum, heart rate variability, intracellular calcium

## Abstract

Theil entropy is a statistical measure used in economics to quantify income inequalities. However, it can be applied to any data distribution including biological signals. In this work, we applied different spectral methods on heart rate variability signals and cellular calcium oscillations previously to Theil entropy analysis. The behavior of Theil entropy and its decomposable property was investigated using exponents in the range of [−1, 2], on the spectrum of synthetic and physiological signals. Our results suggest that the best spectral decomposition method to analyze the spectral inequality of physiological oscillations is the Lomb–Scargle method, followed by Theil entropy analysis. Moreover, our results showed that the exponents that provide more information to describe the spectral inequality in the tested signals were zero, one, and two. It was also observed that the intra-band component is the one that contributes the most to total inequality for the studied oscillations. More in detail, we found that in the state of mental stress, the inequality determined by the Theil entropy analysis of heart rate increases with respect to the resting state. Likewise, the same analytical approach shows that cellular calcium oscillations present on developing interneurons display greater inequality distribution when inhibition of a neurotransmitter system is in place. In conclusion, we propose that Theil entropy is useful for analyzing spectral inequality and to explore its origin in physiological signals.

## 1. Introduction

Biological signals are diverse and of great value as they can be used to understand physiological processes [1,2]. For instance, direct evaluation of signals in the clinic can provide an intuitive description that facilitates diagnostics [3]. Like most signals in the real world, they have irregularities and various shapes, and they are neither purely periodic nor can they be expressed with an analytical formula. These irregularities are signs of the uncertainty in the evolution of the observed physiological processes. Importantly, this uncertainty limits the predictability of the signals analyzed but also carries important information [3,4]. Due to this unpredictability in the temporal domain, some researchers have considered the analysis of the frequency domain. The Fourier transform converts signals from the time domain to the frequency domain, decomposing a signal into multiple periodic components. The discrete Fourier transform is an adaptation used in most scenarios for the digitization of a continuous natural phenomenon in finite samples uniformly spaced. The problem with this approach is that it only provides a discrete description of the phenomenon and does not provide complete information regarding its evolution. It must also be noted that the energy spectrum obtained from the physiological signals is not distributed in a linear way at some frequencies due to the irregularities inherent to these frequencies [3,4]. Thus, there are additional methods that try to characterize the irregularities of digital signals in the frequency domain. One of the preferred methods is based on spectral entropy, analogous to Shannon entropy in information theory [5,6]. Spectral entropy is a function of the irregularity of amplitude and frequency of the power spectrum peaks and it is derived by applying Shannon entropy to power spectra [7,8,9]. Estimations on the frequency grid are firstly divided by the total power, and then, a list of proxies in the form of probabilities, whose sum is one, is obtained. Then, the Shannon entropy formula, which is the negative sum of probability-weighted log probabilities, maps those proxies into a quantity representing the irregularity of energy distribution on the frequency domain. Under this perspective, a flat spectrum has maximal spectral entropy, and the spectrum of a single frequency signal has minimal spectral entropy, which is zero [4]. Spectral entropy has been applied in diverse areas, including endpoint detection in speech segmentation [10] and spectrum sensing in cognitive radio networks [11]. Moreover, it has also served as the base for a famous inductive bias, maximum entropy [12], which is widely adopted for spectrum estimation of some kinds of physiological signals like electroencephalogram (EEG).

Although spectral entropy provides information on the irregularity of a signal, it does not provide information on the origin of spectrum inequalities. This is in line with the fact that any permutation of the values in the frequency spectrum will give the same value of the spectral entropy [4]. That said, it would be reasonable to think that the methods derived from Shannon entropy do not contain information about the location where the greatest irregularity in the spectrum occurs, nor about the changes that occur in the low or high tail of the distribution formed by ordering the frequencies from lowest to highest energy. In conclusion, the frequency components of different intensities in the spectrum close to one or the other, or those components with similar intensity, whether they are far apart in the frequency spectrum or not, are obviated in traditional methods, which in turn are limited to globally capturing the irregularities of the spectrum.

Here, we are introducing Theil entropy for the analysis of the frequency domain of the spectrum. This has been used mainly in the analysis of economic inequalities between various agents [13], along with other indicators such as the Gini index [14,15]. An increase in biological inequality implies the concentration of oscillations around a certain type, meaning that the occurrence of same type oscillations increases. Complementarily, the diversity of oscillation types, as defined by their frequency and energy, decreases. This phenomenon can be clearly appreciated with synchronic discharges observed in the EEG, however this is a more general effect susceptible to analysis in all biological signals. In particular, the application of the Gini coefficient to biological phenomena has recently been explored for the analysis of the electrocardiogram [16], in EEG [17], in the evaluation of diffusion magnetic resonance imaging [18], exploring the variability of the radionic characteristics of lung cancer lesions in non-contrast and contrast-enhanced chest computed tomography images [19], as an unbiased tool for the selection of genes in the analysis of gene expression [20], and in the study of oscillations of intracellular calcium in developing neurons [21]. In these cases, the Gini coefficient, applied to determine the spectral inequality of biological oscillations, has been used as a linear measure of the inequality of the distributions [16,17,21]. However, although the Gini coefficient and Theil entropy are used as indicators of inequality, they are very different when it comes to providing information on the origin of inequality by not setting the same criteria in the distributive analysis [22].

Theil entropy fulfills most of the desirable properties that can be required of inequality indices. That is, being independent of the scale and size of the population and satisfying the principle of transfers of Pigou–Dalton, while also being decomposable [13,23,24,25,26]. More in detail, the β parameter in the Theil entropy equation (Equation (4)) affects the sensitivity of the index to transfers between rich and poor, depending on where is computed in the distribution. Shorrocks showed that as β decreases, the T (β) index is more sensitive to transfers in the lower part of the distribution [13]. Extrapolating this to the frequency spectrum answers the question of how much inequality can be attributed to the differences in low and high energy frequencies.

Previous work on the inequality of the frequency spectrum for biological oscillations [16,17,21] has been limited to describing the inequality of the spectrum without addressing its origin. Among the possible application of these inequality indices, one is to provide information about the causes of inequality, as well as clarifying the relative importance of the contributing elements. In this context, the most widely used analysis is the additive decomposition of the Theil index.

Decomposing an index additively into a series of factors is equivalent to determining which part of the total inequality is attributed to each of those factors [24,27]. One of the most widespread proposals applied in distributive analysis is the one in which inequality is the result of differences within subgroups and of gaps between subgroups [22,28,29]. Formally in 1984, Shorrocks defined the additively decomposable measure into an intragroup component and described the part of the total inequality that is attributable to differences within subgroups [13]. This term is a weighted average of the inequality of the subgroups where the weights depend on the population and the income share, when applied to economics. A second term is the intergroup component and measures the existing inequality due to differences in group mean income. This term represents the inequality that would be observed if the income of each person were replaced by the mean income of each respective subgroup [27].

Extrapolating the above, it would be possible to apply Theil entropy analysis in the subdivision of frequency bands in the spectral analysis of physiological signals from a wide rage. Application of Theil entropy to the analysis of Heart Rate Variability (HRV) and cellular calcium oscillation will transcend the previous analyses of spectral inequality using the Gini Coefficient [16,30]. For these signals the decomposability involves the subdivision of frequencies into homogeneous, exhaustive and mutually exclusive frequency bands, to analyze what part of the total inequality is attributable to each of these bands [24]. Thus, in this article the effectiveness of the Theil Family indices applied on frequency spectra of physiological signals is explored.

## 2. Materials and Methods

### 2.1. Signals and Clinical Datasets

To test the proposed methodology, tests were carried out on both synthetic signals and biological signals; these being the tachogram derived from the RRs of ECG signals and calcium signals captured in developing cortical interneurons.

#### 2.1.1. Synthetic Signals

The signals were created with a component of *DC* (static component) and *AC* (variant component), to which white noise (*WN*) was added to contaminate it. The fixed frequencies were determined in a range that goes from 0.003 Hz to 0.4 Hz, which corresponds to the range of the frequency bands of interest, associated with the analysis of Heart Rate Variability (VLF, LF and HF). The model that was considered for the signals was:

(1) $$y\left(t\right)=AC+DC+WN;\;\mathrm{where}\;AC=4cos\left(2\pi \left(f_{i}\right)t\right),\;\mathrm{DC}=1$$

To know the impact of energy transfers from one frequency to another, the *AC* component was varied and the *DC* and *WN* were kept constant. The signals were constructed by iterating on a vector containing 40 frequency values, equally spaced, between 0.003 and 0.4 Hz. Therefore, 40 signals were obtained each with the maximum energy peak coinciding with a value of the vector as previously explained. The function that was used for the construction of these 40 signals was:

(2) $$y\left(t,f\right)=1+4cos\left(2\pi \left(f_{i}\right)t\right)+1.5\left[\frac{t}{1000}+randn\left(length\left(t\right)\right)\right]$$

where *f* is the vector with 40 values equally spaced between 0.003 Hz and 0.4 Hz, *f_i_* the *i*-th frequency of this vector, $WN=1.5\left[\frac{t}{1000}+randn\left(length\left(t\right)\right)\right]$, *t* is the time vector with 1000 s length and 1000 Hz sampling rate. Matlab function randn was used to generate random numbers.

Likewise, to assess the changes produced in the Theil entropy when varying the exponent, a vector A was constructed with 20 values equally spaced from −1 to 2, iterating the Theil entropy equation (Equation (4)) over *A*.

For the generation of PSDs, the Matlab FFT function was used. For a signal of N points in the time domain, FFT calculates a signal of frequencies of *N* points that ranges from zero to the Nyquist frequencies, therefore the length of the signal limits the resolution of the frequencies. This can be extended by increasing the length of the time series with zeros. This process is known as zero padding and constitutes the *N* argument of the Matlab FFT function. We use *N* = 2048 to obtain a frequency spectrum with 1025 points (*N*/2 + 1).

To obtain the PSD, the Fourier transform was conjugated and normalized dividing it by N. After this process the first *N*/2 values are valid, as the other half is an inverted image and therefore redundant of the first. However, it contains the same power information, which must be considered by multiplying the power spectrum by a factor of two, except for the first and last value.

Spectral leakage is a phenomenon that occurs when the power of the spectrum is calculated using the Fourier transform. This effect can be attenuated using the Hanning window, which is defined by:

(3) $$W_{Hanning}\left(t_{n}\right)=0.5-0.5cos\frac{2\pi t_{n}}{t_{N}},0\leq t_{n}\leq t_{N}$$

**Figure 1:**
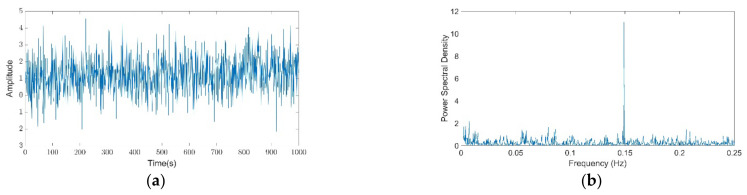
Synthetic signal sample and its computed power spectral density analysis: (**a**) Synthetic signal considering a duration of 1000 s; (**b**) power spectral density (PSD) analysis of synthetic signal.

White noise was computed 1000 times, with the aim of averaging the matrices resulting from the iteration on vectors *F* and *A*, obtaining a single 20 × 40 matrix. Figure 1 shows a signal and its corresponding PSD built under the proposed model, with one *DC* component (invariant component equal to 1) and one *AC* component (variant component) plus *WN* (white noise), for each determined frequency.

#### 2.1.2. RR Intervals

The interbeat intervals (RR-interval signals, the abbreviation of RR referred to the interval between two successive R peaks of electrocardiogram (ECG) signals) were collected for a previous study [16] and were obtained from measurements of RR intervals and databases found at [31]. More in detail, as described previously, thirteen healthy subjects (seven females, six males) aged 19 ± 1.5 years participated in this study. Body mass index (BMI) for participants was 22.3 ± 1.3 kg/m^2^. A-priori power analysis found that this number of participants would yield 80% power at an alpha level of 0.05. All the subjects were non-smokers and had no history of heart disease, systemic hypertension or any other disease. Participants did not take any medications, drugs or alcohol for 12 h preceding the experiment and were advised not to drink any caffeinated beverages on the morning of the study. ECGs were taken in a sitting position and RR intervals were obtained by electrocardiogram during rest (five min) and during mental stress (arithmetic challenge; five min).

#### 2.1.3. Image Intensity Data Collection

Images analyzed in this study were obtained from data sets collected in a previous study [32]. Sample images can be observed in the following link to our recent publication: https://www.mdpi.com/1422-0067/21/21/8013/htm (accessed on 21 Ocober 2021). In more detail, signals were collected using two photon microscopy on brain slices prepared from embryonic mouse brain at the embryonic day 13 (E13). For this purpose, brain slices were loaded with Fluo 4 am and recording of spontaneous activity was performed using culture media on the air liquid interface at 37 °C and 5% CO_2_. In the experimental condition, glycine receptor inhibition was achieved using strychnine at a final concentration of 1 μM, introducing it just before imaging. As per the image analysis, fluorescence intensity measurements were performed using Imagej software V1.49k. Regions of interest (ROI) were defined to circle the entire cytoplasm of the cell and were repositioned in every frame using the ROI manager tool ensuring precise tracking of the cell along the entire time series. Then, mean intensity values were extracted measuring the circled area intensity using the same tool.

### 2.2. Theil Entropy

This article proposes a new method for the analysis of the uniformity of the spectrogram of a signal based on Theil Entropy (TE). This family of indices has been used to measure economic inequality as well as racial segregation in different social settings.

Theil entropy assigns a value to the feature set {*y*_1_, …, *y*_n_}. Taking this into account, it is possible to assign a generalized entropy value to the spectral density. This is possible as it is a mathematical function that assigns an energy value to each frequency that makes up the signal. Therefore, from the analysis of the spectral density, two vectors *X* = [*x*_1_, …, *x_n_*] and *Y* = [*y*_1_, …, *y_n_*] are derived, where *x* is the vector of the frequencies and *Y* is the vector of the spectral density of associated energy. That said, the frequencies (*X)* can be considered as the agents or subjects and the spectral density (*Y*) as the income of each agent. If we establish the spectral density as the so-called characteristics of the n frequencies, we obtain that the Theil entropy would be given by the following formula,

(4) $$TE\left(\alpha \right)=\frac{1}{n\left(a^{2}-a\right)}\sum_{1}^{n}\left[{\left(\frac{y_{i}}{\bar{y}}\right)}^{\alpha }-1\right]\;\mathrm{for}\;\alpha \neq 0,1$$

Since the denominator is equal to 0 in for values of α = 0.1; Using the l’Hopital rule, we obtain:

(5) $$TE\left(0\right)=\frac{-1}{\bar{y}}\sum_{1}^{n}ln\left(\frac{y_{i}}{\bar{y}}\right)$$

(6) $$\mathrm{and}\;TE\left(0\right)=\frac{1}{n}\sum_{1}^{n}\frac{y_{i}}{\bar{y}}ln\frac{y_{i}}{\bar{y}},\;\mathrm{where}\;y=\frac{\sum y_{i}}{n},$$

*y_i_* is the equivalent of the spectral density of the frequency *i*, *α* = {−∞, ∞}, and n is the frequency number in the band. The parameter α summarizes the sensitivity of the index to income differences in different parts of the spectrum. For long values of *α* and positive the generalized entropy is sensitive to changes in the distribution that affect the upper bound of the distribution, with a small and positive alpha it will do so for the lower bound of the distribution. If it is negative, it is sensitive to changes in the distribution that affect the lower bound. It should be noted that when talking about the lower or upper bound of the frequency distribution, it refers to those frequencies with lower or higher energy and not to low or high frequencies.

The Theil index, when *α* = 1, values the frequencies differently, unlike other indices such as Atkinson’s, which assigns the same weight to all agents. In this case, ln (yiy) is a weighting factor that assigns a negative and proportionally greater weight to frequencies that have lower than average energy and positive and proportionally lower ones to those that have higher than average energy. While the lower limit of GE (*α*) is always zero, perfect equality, the upper limit varies with the value of *α*. For *α* > 0 T (*α*) presents an upper bound that depends on n, the maximum value for the Theil index being equal to ln (*n*); on the contrary, when *α* ≤ 0 the family of Theil indices is not bounded superiorly.

The total inequality can be written as the sum of the inequality within the *GE_W_* bands (*α*) and the inequality between the *TE_B_* bands (*α*), where the former is the weighted sum of the inequalities within each band:

(7) $$TE\left(\alpha \right)=TE_{W}\left(\alpha \right)+TE_{B}\left(\alpha \right)$$

(8) $$TE\left(\alpha \right)=\overset{G}{\underset{j=1}{\sum }}TE_{j}\left(\alpha \right){\left(\frac{\bar{y_{j}}}{\bar{y}}\right)}^{\alpha }+\frac{1}{\alpha^{2}+\alpha }\left[\overset{G}{\underset{j=1}{\sum }}g_{j}\left(\frac{\bar{y_{j}}}{\bar{y}}\right)-1g_{j}\left(\frac{\bar{y_{j}}}{\bar{y}}\right)-1\right]$$

(9) $$TE\left(0\right)=\overset{G}{\underset{j=1}{\sum }}TE_{j}\left(0\right)+\left[\overset{G}{\underset{j=1}{\sum }}g_{j}ln\left(\frac{\bar{y}}{\bar{y_{j}}}\right)\right]$$

(10) $$TE\left(1\right)=\overset{G}{\underset{j=1}{\sum }}TE_{j}\left(1\right)g_{j}\left(\frac{\bar{y_{j}}}{\bar{y}}\right)+\left[\overset{G}{\underset{j=1}{\sum }}g_{j}\left(\frac{\bar{y_{j}}}{\bar{y}}\right)ln\left(\frac{\bar{y}}{\bar{y_{j}}}\right)\right]$$

where *j* refers to band, *GE_j_* is the inequality in band *j* and *g_j_*, which represents the number of frequencies in band *j* of the total number of frequencies.

### 2.3. Spectral Density Analysis

The PSD presents spectral power density of a time series as a function of frequency. Therefore, PSD estimates can give information about the amount of power in which certain frequencies contribute to a time series. Estimating the PSD can be performed using many methods, however methods based on Fast-Fourier Transform (FFT) and autoregressive (AR) modeling are perhaps the most popular. Classical power spectrum estimates developed by Bartlett (1948), Blackman and Tukey (1958), and Welch (1967) are examples of methods based on FFT [33]. As the FFT makes no assumptions on how the data are generated the classical methods are often referred to as non-parametric. The AR power spectrum methods do make assumptions and are therefore called parametric. Contributing to the popularity of the FFT based estimates are their simplicity, broad understanding, and ease of computation using modern computers and software. However, both FFT and AR based PSD estimates have prerequisites that are seldom, if ever, met by biological signals such as cardiac RR series [33]. Both methods require the analyzed time signal to be stationary and evenly sampled, which is inherently not the case with RR series [34]. Traditional methods require the transformation of the original non-uniformly spaced electrocardiogram RR interval series into regularly spaced ones using interpolation or other approaches [35]. Consequently, other methods such as the Lomb–Scargle periodogram have become popular as they do not require resampling [33,36,37]. The Lomb–Scargle (L-S) method uses the raw original RR series, avoiding different artifacts introduced by traditional spectral analysis methods [35]. Despite the aforementioned limitations of FFT and AR based PSD estimates, they are widely used in signal processing.

As mentioned before, the Theil index family is a mathematical function that assigns an energy value to each frequency that makes up the signal. However, the spectral density analysis is calculated using several methods.

#### 2.3.1. Welch Periodogram

To understand Welch’s periodogram one must first understand the discrete Fourier transform (DFT), the basic periodogram, and the modified periodogram. The *N*-point DFT of a random variable *X*(*n*) is given by:

$$DFT_{x}\left(f\right)=\overset{N-1}{\underset{n=0}{\sum }}X\left(n\right)e^{-i2\pi fn}$$

Practical computations of the *DFT* use the *FFT* for speed advantages. The periodogram, extension of the *DFT*, is a basic method of estimating power spectral density of a time series and is given by:

$$P\left(f\right)=\frac{1}{N}\vee \sum_{n=0}^{N-1}X\left(n\right)e^{-i2\pi fk/L}\vee ²;\;\;k=0,\;1,\;...,\;L-1$$

Reducing spectral leakage of the periodogram can be accomplished by incorporating a weighted windowing function *w*(*n*), e.g., Hamming and Hanning, to the input series. Data near the edges of the time series are given less weight compared to data nearer the center. Thus, the modified periodogram is given by:

$$P\left(f\right)=\frac{1}{MU}\sum_{n=0}^{M-1}X\left(n\right)w\left(n\right)e^{-i2\pi fn}\vee^{2},\;\mathrm{where}\;U=1/M\sum_{n=0}^{M-1}w²\left(n\right)$$

Finally, in an effort to reduce the variance of the periodogram estimation, the Welch method separates the data series into *N* overlapping segments. As with the modified periodogram the Welch method applies a weighting window to reduce spectral leakage, however, weighting is applied to each segment. Finally an averaged PSD is calculated using all segments [33]. Power spectral density by the Welch periodogram is given by:

$$P_{W}\left(f\right)=\frac{1}{N}\overset{N-1}{\underset{i=0}{\sum }}P_{M,i}\left(f\right)$$

where *P_M,i_* (*f*) is the *i*th modified periodogram from data series.

#### 2.3.2. Lomb–Scargle Periodogram

Lomb–Scargle periodogram (LSP) method of estimating PSD does not require resampling. The LSP only uses available data. Conceptually LSP estimates the frequency spectrum by performing a least squares fit of sinusoids to the data. Unlike Welch’s periodogram, weighted windowing functions are not applied to data in LSP as standard weighting methods cannot be applied to unevenly sampled data.

The LSP of a non-uniformly sampled, real-valued data sequence *X* of length *N* for arbitrary times *t_n_* is defined by

$$P_{LS}\left(f\right)=\frac{1}{2\sigma 2}\frac{{\left[\sum_{n=1}^{N}\left(X\left(t_{i}\right)-\bar{X}\right)cos\left(2\pi \left(t_{n}-\tau \right)\right)\right]}^{2}}{\sum_{n=1}^{N}cos^{2}\left(2\pi f\left(t_{n}-\tau \right)\right)}+\frac{{\left[\sum_{n=1}^{N}\left(X\left(t_{i}\right)-\bar{X}\right)sin\left(2\pi \left(t_{n}-\tau \right)\right)\right]}^{2}}{\sum_{n=1}^{N}sin^{2}\left(2\pi f\left(t_{n}-\tau \right)\right)}$$

#### 2.3.3. Burg Periodogram

Autoregressive spectral estimation methods differ from non-parametric methods in that they attempt to model the data instead of estimating the PSD directly [35]. Several modeling methods exist for AR spectrum estimation, however the Burg method is one of the most used in HRV analysis.

The power spectrum of a pth order autoregressive process is given by

$$P_{Burg}\left(f\right)=\frac{1}{f_{s}}\frac{e_{p}}{1+\sum_{k=1}^{p}a_{p\left(k\right)e-2\pi jkf/f_{s}}\vee^{2}}$$

where *ε_p_* is the total least square error, *f_s_* is the sample rate, and ap are the Burg AR model parameters [38] suggests that a model order of *p* = 16–20 is a sound choice for physiological signal in a human resampled at 2–4 Hz.

## 3. Results

### 3.1. Theil Entropy Analysis of Synthetic Signals Shows the Potential of This Method to Account for Inequality Distribution of Power Spectra

**Figure 2:**
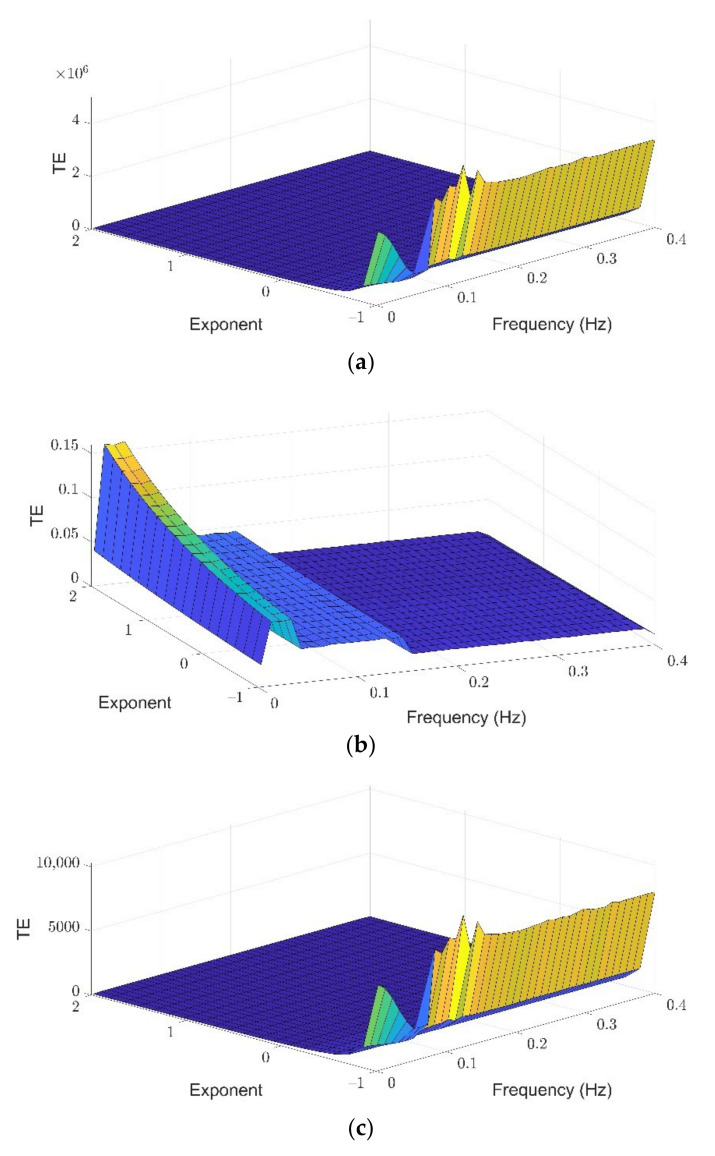
Three-dimensional graphs showing Theil entropy values for synthetic signals. Values were obtained by averaging the 1000 matrices generated and are shown as total and decomposed. (**a**) Total Theil entropy that can be decomposed into (**b**) an inter-band compontent (*TE_B_*), inequality not from within each frequency band but among them, and (**c**) an intraband component (*TE_W_*), inequality within each frequency band.

To get a sense of how Theil entropy could aid in providing an improved description of inequality distribution of power spectra obtained from biological signals, we computed synthetic signals and analyzed their behavior. This allowed us to know the change that occurs in the values of Theil entropy due to the transfer of energy within the same band (TI) and between the bands (TB). In each case, Theil entropy graphs show how Theil entropy values from synthetic signals change as the energy peak moves from the low frequencies to the higher frequencies (Figure 2).

In more detail, Theil entropy analysis performed on synthetic signals showed that the intra-band component is the one that contributes the most to the total value, this means that the origin of the spectral inequality is found in the energy difference that exists within each band and not in the differences between the bands (Figure 2). Likewise, it shows how the Theil entropy value increases as the exponent (alpha) passes through the most negative values. Moreover, the values change if the exponent is kept constant at −1 and the energy peak varies. In this case (when alpha = −1) the index is higher when traveling through high frequencies. If we eliminate the alpha values < −1, we can better appreciate the changes that occur when varying the exponent for positive values, which we have not seen before, since the negative alpha values are very high. For alpha values > 0, Theil entropy tends to grow, and no noticeable differences are observed as the energy peak passes from low to high frequencies.

**Figure 3:**
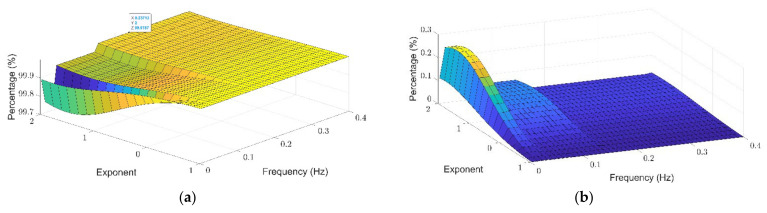
Decomposition of Theil entropy for synthetic signals. Theil entropy is shown decomposed into its two additive components and expressed as a percentage. (**a**) Intra-band Theil Entropy, (**b**) Inter-band Theil Entropy.

To observe the behavior of the two additive components of Theil entropy, the calculated value was decomposed and expressed as percentage (Figure 3). As expected, it was found that the intra-band component contributed a greater weight to the spectral inequality. The decomposition showed a clear division of the frequency bands, increasing the intragroup component as the energy peak moves from low to higher frequencies. Variations are more evident for positive alpha values.

### 3.2. Theil Entropy Effectively Captures Inequality Distribution of Heart Rate Variations in Response to Stress

**Figure 4:**
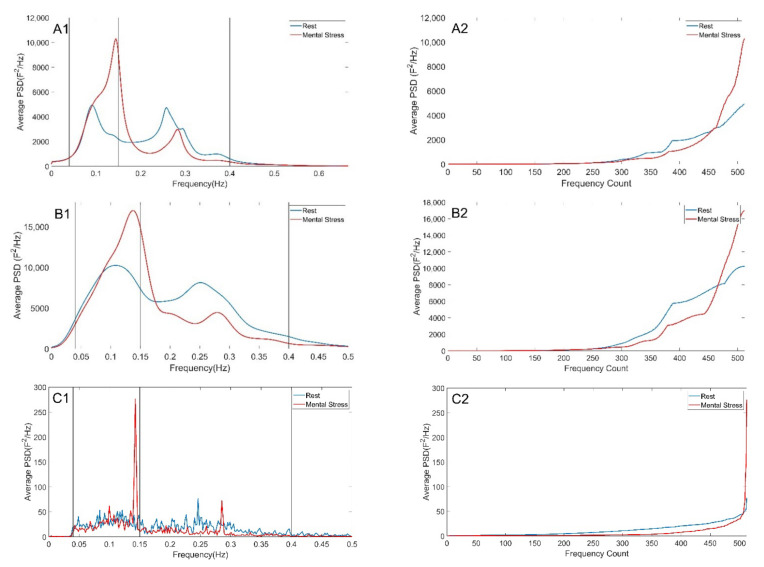
Average spectrogram of all subjects according to the three methods applied. Upper raw shows Burg (**A1**,**A2**), middle raw shows Welch (**B1**,**B2**), and lower raw shows Lomb–Scargle (**C1**,**C2**) methods in baseline stress. Left (**A1**–**C1**): general spectrum. Right (**A2**–**C2**): sorted spectrum according showing in the x axis frequency counts according to their energy from lower to higher. For the ordered spectrum frequency range was divided in 512 bins.

To evaluate the applicability of the additive decomposition of the Theil index to the analysis of physiological signals, HRV data sets in control conditions and under stress were analyzed. Power spectra were calculated using Burg, Welch and Lomb–Scargle spectral methods (Figure 4). Figure 4 shows the average spectrum calculated from the spectrograms of each of the subjects, where blue represents the basal state and red is the spectrum in the mental stress state. A side of the spectrum is shown the average spectrum ordered from lowest to highest energy, which guides us on the distribution of the lower bound and top of the spectrum. In the three spectral methods it can be observed that the maximum energy peak is found in the LF, and there is also a second lower energy peak in the HF. In addition, it is shown that there is greater energy during mental stress. Looking at the ordered spectra, we can see that the Lomb method is smoother and that the greatest irregularities are found in the upper bound of the curves.

In relation to Theil analysis, while the Lomb–Scargle method showed more significant results than the rest, exponents 1 and 2 showed the greatest changes. As can be seen in Table 1, Theil entropy values for exponent -1 are high using the three methods. In turn, exponent 1 provided the lowest values of the Theil index, as well as a larger effect size in those values that were significant (Table 1). The analysis performed using the additive decomposition of the Theil index for each of the selected spectral methods is shown in Table 2, Table 3 and Table 4. On these, it is evident that the inter-band component yielded more significant results. This component quantifies the total inequality part attributed to the differences between the bands disregarding the distribution of energy within the same band. Table 2 shows the analysis when the decomposition of the signal was performed using the Burg method. If we analyze the Theil entropy using the global spectrum and the spectrum of frequencies between 0.004 and 0.4 Hz, the Theil entropy of the global spectrum is greater than that of the reduced spectrum. Similarly, the table shows that the inequality is greater in stress than in the baseline with significant differences (Exp −1, 0, 2). Focusing on the additive decomposition, the component that weights the most is the intra-band. However, the inter-band component shows significant differences in stress and baseline, being significantly higher in stress with a large effect size. The fact that there are significant differences with the four exponents suggests that this decrease in inequality is evident both in the upper and lower bounds of the distribution. Decomposing the intra-band component, the observable inequality is due to the differences in the distribution of high frequencies (HF) in both states, comparatively.

Unlike the previous method, the Welch’s method shows that the inequality attributable to low frequencies (LF) shows significant differences in the states, being greater in stress (Table 3). The inter-band component shows significant differences between the two states. However, the intra-band component is the one that contributes the most to existing inequality. It should be noted that for -1 exponent that assigns greater weight to the lower bound of the distribution, the opposite occurs, and the observable difference is explained by the differences between the frequency bands (Inter).

Table 4 shows the results of application of the Lomb–Scargle method. With this analysis we can see that inequality is greater at the lower end of the spectrum. Focusing on exponents zero, one, and two; although the inequality is relatively small, there are significant differences, being greater in stress. The same occurs with the inter- and intra-band components. The additive decomposition shows that the inequality of the high frequencies contributes more to the total inequality, and this inequality is significantly higher in stress. In the LF band these differences are significant in the upper bound, whereas in the HF they are so in the lower bound.

The analysis of the HRV signals of Stress-baseline using Theil entropy and analyzing the results of the three periograms indicated that in mental stress, inequality increases significantly with respect to the basal state. This increase in inequality has its origin in the intra-band component, however the contribution of the inequality of the bands differs depending on whether we assign a greater weight to the upper or lower bound. In the case of the lower bound (exp −1), the VLF band has a greater weight, and in the upper bound (exp 2), where the frequencies close to the peak are found, the inequality in the LF band has a greater contribution.

### 3.3. Theil Entropy Analysis Reveals Inequality Distribution of Calcium Oscillations in the Developing Brain

**Figure 5:**
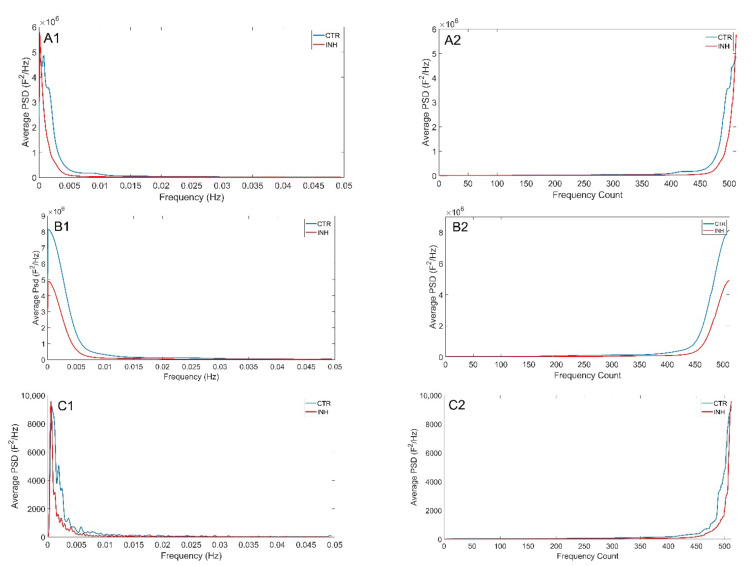
Average spectra of spontaneous calcium oscillation in inhibitory interneurons. Burg (**A1**,**A2**), Welch (**B1**,**B2**), Lomb (**C1**,**C2**) methods were applied to raw data and are presented in each raw from top to bottom. (**A1**–**C1**) disordered spectrum. (**A2**–**C2**) ordered spectrum showing in the x axis frequency counts from lower to higher energies. For the ordered spectrum frequency range was divided in 512 bins.

To broaden the application of the proposed analytical method, we studied intracellular calcium variations that occur spontaneously in interneurons during embryonic cerebral cortical development in mice. Interneurons of the cerebral cortex were analyzed in the early stages of development in control condition and under inhibition by blocking glycine receptors with strychnine. Applicability of the Theil family indices for studying the degree of inequality in calcium oscillations was assessed on spectra generated by the three different methods used before (Figure 5). The average spectra using the three spectral methods in the control and inhibited interneurons shows that the highest energy is found at the lower frequencies and that this energy is higher in the control neurons compared to the inhibited cells.

More importantly, application of the Theil index to the calcium spectra shows greater inequality in the lower bound, where the exponent −1 registers the highest values in the 3 methods (Table 5). In inhibited cells, inequality increases except for the lower bound, which decreases, although these differences are only significant for the upper bound. In other words, if the frequencies with higher energy between the two states are analyzed, the inequality is greater in the inhibited cells with significant results and a large effect size.

## 4. Discussion

The analysis of the spectrogram of biological signals is useful to understand various biological phenomena. The present study used Theil entropy in the analysis of inequality of the spectrogram of biological signals. From this analysis we conclude that: (1) the suggested spectral decomposition method to analyze spectral inequality is the Lomb–Scargle; (2) the exponents that provide the most information to describe the spectral inequality are zero, one, and two; (3) the additive decomposition of generalized entropy provides important information about the origin of this, with the intra-band component being the one that contributes the most to total inequality; (4) in the state of mental stress inequality increases with respect to the state of rest; (5) in developing interneurons, spectral inequality is greater when there is inhibition of calcium dynamics by blocking glycine receptors.

Among the spectral methods used, the Lomb–Scargle method has several properties that make it a more attractive alternative than the others for the study of spectral inequality in biological signals [39]. One of these properties is that it can be used in stochastic and irregularly sampled signals, such as RR signals [40]. The Lomb–Scargle periogram reduces distortions or errors that may result from any interpolation process performed by other methods [41]. In turn, the Welch and Burg methods have a smoothing effect that limits the resolution of the frequencies. Although this can be beneficial in the case of outliers in the time series [35], this effect does not exist in the Lomb–Scargle periogram allowing better frequency resolution and therefore peaks and valleys are exhibited at the most distinctive frequencies. Additionally, unlike methods where the highest concentration of energy occurs around the peaks, the Lomb–Scargle periogram is more accurate when distributing the energy [40]. This phenomenon can be seen in Figure 4, where the frequencies with higher energy belong to the band where the energy peak is found in the Burg and Welch methods, but not in the Lomb–Scargle method, where the energy peaks in a more specific frequency range allowing higher resolution by not smoothing the spectrum.

Jenkins SP [42] indicated that for values of the exponent outside the interval [−1, 2] the generalized entropy would be influenced highly by a small number of frequencies with very low energy or with very high energy. In our work, it was found that outside these limits the entropy values were very large. We also obtained that, although the use of the value −1 is recommended for values of the lower bound of the distribution, the entropy values were too large in some of the spectrograms used. In addition, we found that the generalized entropy measures are not bounded superiorly for exponent values less than zero. However, for small positive exponent values close to zero these measures would be more sensitive to the changes that occur in the lower bound of the distribution [42]. One of the limitations of our study was not using values between zero and one that could explain these changes in the lower bound and that would be bounded by ln(n) as opposed to −1. Another limitation was, for reasons of simplicity, the use in the simulation of a single energy peak in the frequencies of the spectrum as this was a first approach.

As we are proposing, one of the advantages of Theil entropy, in comparison with indices such as Gini’s, is that it can be additively decomposed, providing information on the origin of inequality. Accordingly, the subdivision into frequency bands of the HRV signals spectrogram showed that the intra-band component was the one that contributed the most to the total inequality. Despite that, there are differences regarding the exponent used, with exponent −1 showing a similar contribution to the two components and exponent 2 showing that the intra-band contributes more than 90% to the total inequality. This means that if we analyze the frequencies with lower energy, more than half of the inequality can be explained by the differences between the frequency bands, while if we analyze the frequencies with higher energy, inequality is almost entirely explained by differences in the bands. More in detail, inequality is almost entirely explained by differences between the frequencies of the LF band. The existence of the energy peak in the LF frequency band explains this phenomenon when calculating the Theil entropy with the exponent 2.

An increase in Theil entropy means that the energy is concentrated in few frequencies. This increase could have occurred in the upper or lower side of the distribution, being reflected in the exponent used. It is well known in the study of HRV that the HF band reflects parasympathetic activity while the LF reflects both parasympathetic and sympathetic activity [30,43,44,45,46]. As can be seen in the analysis of HRV, during mental stress the energy peak in the LF is greater than the energy peak that occurs in the basal state, this would explain the increase in spectral inequality by concentrating the total energy in a few frequencies. There are studies investigating the factors that contribute to the energy of LF. Roach et al. [47] reported that 75% of the energy contribution of LFs is due to fluctuations called ripple and that this is probably due to the functions of arterial baroreceptors. Reyes del Paso et al. [48] showed that there is a strong association between baroreflex activity and mental stress. A previous study of spectral inequality using the Gini coefficient showed an increase in inequality in the state of mental stress and that the greatest inequality is concentrated in the LF frequency band [16]. These results agree with ours, as the total inequality increases significantly in the state of mental stress due to inequalities in the LF range. Focusing on the upper bound, that reflects the frequencies with higher energy where the energy peak occurs, it can be seen how Theil entropy increases significantly in mental stress, explaining more than 90% of the observed inequality. In the lower bound, the opposite occurs and the LF only contributes with 3% to the inequality within the bands, while the HF contributes to most of the inequality in the state of mental stress. This is due to the HF band being the one with the highest amount of low energy frequencies.

As it has been mentioned, this research shows an increase in the inequality in the state of mental stress at the expense of inequality within the bands. During the state of mental stress, it is possible that a healthy cardiovascular system generates more LF oscillations especially at frequencies close to 0.1 Hz. This possibility is supported by Bates who evaluated the real-time changes of the spectrum of RR intervals in response to placebo and alcohol. Bates et al. [49] suggested that under the effects of alcohol or other adverse conditions, one of the main adaptations includes maintaining slow oscillations at the expense of fast ones. This is in accordance with the results obtained in our work, where not only did global inequality increase in stress, but so did those of the intra-band and inter-band components, and LF was the band where the most significant differences occurred.

Complementarily, Theil entropy analysis in calcium signals of developing neurons showed that inequality increases significantly at low frequencies close to the energy peak, when comparing neurons under inhibition with control cells. In contrast, at the lower end of the distribution inequality tends to decrease, however this decrease is not significant. Therefore, we hypothesize that inequality increases in inhibited neurons as there is greater synchronization, with calcium oscillating at very similar frequencies where the energy peak occurs. Peak energy is more unequal in inhibited neurons compared to control neurons; this means that there is a higher concentration of energy in this area. For this case, one of the limitations of the study is that the origin of the inequality cannot be explored using the additive decomposition property of Theil entropy due to the non-existence of frequency bands in the spectrum.

Finally, for the study of spectral inequality, the Gini index has been used in biological signals [21]. Although the Gini index is easier to interpret, since it has values between zero and one and is easier to calculate, Theil entropy has certain advantages over Gini. For example, there are distributions that, using the Gini index, are not comparable when the two Lorenz curves intersect as the Lorenz dominance criterion is not met [50,51]. Another limitation of the Gini index resides in the fact that two energy distributions can have the same value of the Gini, having the same area, and being very different distributions [52]. Another disadvantage lies in the additive decomposition of the index that does not allow us to explore the origin of the inequality in the frequency bands and whether these are attributed to inequalities within the bands or between the bands [14]. Theil entropy overcame the limitations of the Gini index on this aspect, as it was additively decomposable. Likewise, the use of the exponent allowed us to focus our analysis on frequencies close to the energy peak or those far from the energy peak with a lower power.

## 5. Conclusions

From the analysis of the inequality of the spectrogram of biological signals using Theil entropy, it is concluded that the exponents that provide the most information to describe the spectral inequality are zero, one, and two, being the Lomb–Scargle method suggested to analyze the spectral inequality. The additive decomposition of the Theil entropy provides important information about the origin of inequality, being the intraband component that contributes the most to the total inequality. In the state of mental stress, inequality increases with respect to the state of rest. Inhibition of glycinergic neurotransmission during brain development affected calcium oscillations increasing their spectral inequality when comparing with control neurons. More generally, Theil entropy is useful in the analysis of spectral inequality and the exploration of its origin in biological signals.

## Figures and Tables

**Table 1:** Theil entropy applied to the global spectrum of heart rate obtained with the Burg, Welch and Lomb–Scargle periogram. Results of the analysis of heart rate in normal conditions and under stress is shown with standard deviation and coefficient of variation values for the exponents −1, 0, 1 and 2. X: average value, SD: standard deviation, CV: Coefficient of variation, *p*: *p*-value, RB: rank biserial correlation [effect size).

Method	Exponent	Rest			Mental Stress			*p*	RB
		X	SD	CV	X	SD	CV		
Burg	−1	328.48	430.19	130.97	83.45	71.6	85.8	0.99	0.74
	0	2.57	0.63	24.51	2.37	0.34	14.41	0.89	0.39
	1	1.2	0.22	18.5	1.32	0.19	14.59	0.05 *	−0.54
	2	2	0.86	43.01	2.34	0.79	33.83	0.21	−0.28
Welch	−1	189.03	171.03	90.48	56.98	40.77	71.55	0.99	0.69
	0	2.48	0.63	25.45	2.25	0.63	28.01	0.91	0.41
	1	1.07	0.19	17.61	1.21	0.13	11	0 *	−0.89
	2	1.38	0.44	32.19	1.83	0.39	21.44	0 *	−0.85
Lomb-Scargle	−1	780.01	624.44	80.06	33,059.8	100,850.69	305.06	0.42	−0.08
	0	1.45	0.44	30.07	1.85	0.5	26.96	<0.001 *	−0.96
	1	1.09	0.35	31.9	1.6	0.33	20.84	<0.001 *	−1
	2	2.29	1.35	59.01	6.02	2.88	47.9	<0.001 *	−1

^*^ Statistically significant differences.

**Table 2:** Additive decomposition of Theil entropy for the Burg periogram analysis of heart rate. Decomposition of the signal was performed using the autoregressive method. TI: Intra-band, TB: Inter-band.

Theil	Exp	Rest			Mental Stress			*p*	RB
		X	SD	CV	X	SD	CV		
VLF	−1	0.001	0.0004	40.00	0.001	0.0005	50.00	0.5	−0.011
	0	0.0006	0.0002	33.33	0.0007	0.0004	57.14	0.271	−0.209
	1	0.0004	0.0003	75.00	0.0007	0.0008	114.29	0.42	−0.077
	2	0.0002	0.0003	150	0.0009	0.002	222.2	0.294	−0.187
LF	−1	0.005	0.003	60.00	0.005	0.003	60.00	0.658	0.121
	0	0.018	0.009	50.00	0.026	0.016	61.54	0.122	−0.385
	1	0.067	0.043	64.18	0.132	0.085	64.39	0.029 *	−0.604
	2	0.282	0.250	88.65	0.691	0.474	68.59	0.024 *	−0.626
HF	−1	0.054	0.043	79.63	0.084	0.058	69.05	0.007 *	−0.758
	0	0.102	0.062	60.78	0.107	0.054	50.47	0.249	−0.231
	1	0.226	0.171	75.66	0.143	0.061	42.66	0.892	0.385
	2	0.550	0.510	92.72	0.203	0.096	47.29	0.966	0.560
TI	−1	0.06	0.043	71.67	0.09	0.057	63.33	0.009 *	−0.736
	0	0.121	0.064	52.89	0.133	0.056	42.11	0.207	−0.275
	1	0.294	0.167	56.80	0.276	0.121	43.84	0.473	−0.033
	2	0.832	0.444	53.36	0.895	0.522	58.32	0.368	−0.121
TB	−1	0.086	0.047	54.65	0.139	0.046	33.09	<0.001 *	−0.912
	0	0.059	0.045	76.27	0.105	0.035	33.33	0.002 *	−0.868
	1	0.05	0.049	98.00	0.099	0.038	38.38	0.002 *	−0.868
	2	0.110	0.060	54.54	0.110	0.047	42.72	0.002 *	−0.846
TI + TB	−1	0.146	0.081	55.48	0.229	0.094	41.05	<0.001 *	−0.978
	0	0.179	0.081	45.25	0.238	0.085	35.71	0.004 *	−0.802
	1	0.344	0.161	46.80	0.375	0.133	35.47	0.271 *	−0.209
	2	0.883	0.439	49.71	1.005	0.527	52.43	0.368 *	−0.121

* Statistic significant differences.

**Table 3:** Additive decomposition of Theil entropy for the Welch periogram analysis of heart rate. TI: Intra-band, TB: Inter-band.

Theil	Exp	Rest			Mental Stress			*p*	RB
		X	SD	CV	X	SD	CV		
VLF	−1	0.017	0.006	35.29	0.019	0.011	57.89	0.42	−0.077
	0	0.011	0.003	27.27	0.01	0.002	20.00	0.607	0.077
	1	0.007	0.002	28.57	0.01	0.008	80.00	0.368	−0.121
	2	0.005	0.004	80.00	0.016	0.028	175.00	0.368	−0.121
LF	−1	0.001	0.0007	70.00	0.003	0.002	66.67	0.02 *	−0.648
	0	0.005	0.003	60.00	0.013	0.01	76.92	0.004 *	−0.802
	1	0.019	0.017	89.47	0.064	0.047	73.44	0.003 *	−0.824
	2	0.085	0.108	127.06	0.318	0.234	73.58	0.005 *	−0.78
HF	−1	0.04	0.033	82.50	0.062	0.043	69.35	0.005 *	−0.78
	0	0.073	0.038	52.05	0.082	0.039	47.56	0.207	−0.275
	1	0.151	0.083	54.97	0.114	0.05	43.86	0.927	0.451
	2	0.334	0.236	70.66	0.169	0.087	51.48	−0.78	0.648
TI	−1	0.059	0.031	52.54	0.083	0.038	45.78	0.002 *	−0.868
	0	0.089	0.04	44.94	0.105	0.041	39.05	0.108	−0.407
	1	0.177	0.087	49.15	0.188	0.085	45.21	0.446	−0.055
	2	0.424	0.234	55.19	0.503	0.3	59.64	0.249	−0.231
TB	−1	0.072	0.045	62.50	0.131	0.032	24.43	<0.001 *	−0.956
	0	0.053	0.044	83.02	0.099	0.026	26.26	<0.001 *	−0.912
	1	0.047	0.047	100.00	0.093	0.03	32.26	0.002 *	−0.868
	2	0.3	0.057	19.00	0.101	0.036	35.64	0.003 *	−0.824
TI + TB	−1	0.131	0.071	54.20	0.214	0.064	29.91	<0.001 *	−0.956
	0	0.142	0.068	47.89	0.203	0.059	29.06	<0.001 *	−0.956
	1	0.224	0.092	41.07	0.28	0.087	31.07	0.013 *	−0.692
	2	0.473	0.23	48.63	0.604	0.291	48.18	0.108	−0.407

* Statistically significant differences.

**Table 4:** Additive decomposition of Theil entropy for the Lomb–Scargle periogram analysis of heart rate variation.

Theil	Exp	Basal			Mental Stress			*p*	RB
		X	SD	CV	X	SD	CV		
VLF	−1	0.309	0.341	110.36	0.17	0.105	61.76	0.936	0.473
	0	0.035	0.021	60.00	0.04	0.023	57.50	<0.001 *	−1
	1	0.01	0.015	150.00	0.022	0.045	204.55	0.207	−0.275
	2	0.004	0.009	225.00	0.031	0.099	319.35	0.227	−0.253
LF	−1	0.018	0.008	44.44	0.014	0.005	35.71	0.945	0.495
	0	0.048	0.009	18.75	0.076	0.02	26.32	0.473	−0.033
	1	0.152	0.091	59.87	0.416	0.13	31.25	<0.001 *	−1
	2	0.599	0.707	118.03	2.345	0.955	40.72	<0.001 *	−1
HF	−1	0.121	0.041	33.88	0.168	0.081	48.21	0.007 *	−0.758
	0	0.173	0.036	20.81	0.177	0.048	27.12	0.02 *	−0.648
	1	0.264	0.101	38.26	0.196	0.05	25.51	0.945	0.495
	2	0.431	0.291	67.52	0.229	0.094	41.05	0.971	0.582
TI	−1	0.449	0.352	78.40	0.352	0.092	26.14	0.58	0.055
	0	0.256	0.056	21.88	0.292	0.067	22.95	<0.001 *	−0.934
	1	0.426	0.426	100.00	0.634	0.152	23.97	<0.001 *	−1
	2	1.034	0.678	65.57	2.605	0.931	35.74	<0.001 *	−1
TB	−1	0.345	0.27	78.26	0.305	0.074	24.26	0.554	0.033
	0	0.098	0.047	47.96	0.165	0.035	21.21	0.002 *	−0.868
	1	0.063	0.051	80.95	0.143	0.044	30.77	<0.001 *	−0.978
	2	0.059	0.064	108.47	0.157	0.055	35.03	<0.001 *	−0.978
TI + TB	−1	0.793	0.594	74.91	0.657	0.14	21.31	0.554	0.033
	0	0.354	0.083	23.45	0.457	0.094	20.57	<0.001 *	−0.956
	1	0.488	0.157	32.17	0.777	0.182	23.42	<0.001 *	−1
	2	1.094	0.731	66.82	2.763	0.978	35.40	<0.001 *	−1

* Statistic significant differences.

**Table 5:** Theil index applied to spontaneous calcium oscillation in inhibitory interneurons. Burg, Welch, Lomb methods were applied to raw data.

Method.	Exp	Control			Under Inhibition			*p*	RB
		X	SD	CV	X	SD	CV		
Burg	−1	13.442	16.813	125.08	9.652	5.795	60.04	0.294	0.084
	0	2.032	0.878	43.21	2.267	0.591	26.07	0.148	−0.161
	1	2.01	0.727	36.17	2.386	0.602	25.23	0.016 *	−0.326
	2	8.084	5.482	67.81	10.794	6.094	56.46	0.027 *	−0.292
Welch	−1	9.076	11.91	131.23	5.595	3.658	65.38	0.562	0.023
	0	1.73	0.771	44.57	1.749	0.48	27.44	0.342	−0.063
	1	1.481	0.472	31.87	1.643	0.33	20.09	0.116	−0.183
	2	3.188	1.374	43.10	3.769	1.087	28.84	0.042 *	−0.262
Lomb	−1	4.791 × 10 ^25^	2.443 × 10 ^26^	500.99	9.448 × 10 ^24^	1.616 × 10 ^25^	171.04	0.643	0.054
	0	2.256	0.719	31.87	2.366	0.418	17.67	0.148	−0.161
	1	2.003	0.591	29.51	2.282	0.431	18.89	0.013 *	−0.337
	2	8.357	5.095	60.97	11.272	4.813	42.70	0.004 *	−0.398

* Statistically significant differences.

## Data Availability

The datasets used or analyzed during the current study are available from the corresponding author on reasonable request.
